# Melatonin Supplementation Increases Oocyte Recovery and Quality Associated with Altered Follicular Steroidogenesis and Redox Status in Dairy Cows

**DOI:** 10.3390/antiox15091101

**Published:** 2026-08-31

**Authors:** Wenkui Ma, Qianru Chen, Depeng Yin, Pengyun Ji, Liming Liu, Xihe Li, Bingyuan Wang, Lu Zhang, Guoshi Liu

**Affiliations:** 1 State Key Laboratory of Animal Biotech Breeding, College of Animal Science and Technology, China Agricultural University, Beijing 100193, China; mawenkui@cau.edu.cn (W.M.); qrchen@cau.edu.cn (Q.C.); yindepeng@cau.edu.cn (D.Y.); jipengyun@cau.edu.cn (P.J.); wangbingyuan@cau.edu.cn (B.W.); 2 The State Key Laboratory of Reproductive Regulation and Breeding of Grassland Livestock, Inner Mongolia University, Hohhot 010070, China; nmgdxllm@163.com (L.L.); lixh@imu.edu.cn (X.L.)

**Keywords:** dairy cow, ovum pick-up (OPU), melatonin, oocyte quality, follicular microenvironment, steroidogenesis

## Abstract

Ovum pick-up (OPU) enables high-frequency oocyte retrieval from selected dairy cow donors, facilitating favorable genetic progression. However, oocytes obtained via OPU and subjected to in vitro maturation frequently suffer from severe oxidative stress, mitochondrial dysfunction, and DNA damage, resulting in lower pregnancy rates compared to those of in vivo-derived embryos. Melatonin (MT), as a potent antioxidant, may exert beneficial effects on these OPU-retrieved oocytes. In the current study, by combining in vitro and in vivo experiments, the protective effects and potential molecular mechanisms of MT on disrupted oocyte quality and development caused by OPU were systemically investigated. By integrating oocyte quality assessment, follicular fluid metabolomics, and blastocyst transcriptomics, the results showed that subcutaneous administration of MT to cows prior to OPU significantly increased the number of high-quality Grade A oocytes and reduced the number of low-quality Grade D oocytes compared to the control. These improvements were accompanied by reduced local and systemic oxidative stress, enhanced glutathione metabolism, and lower serum progesterone concentrations at the measured time points, coinciding with a more favorable endocrine environment for follicular development. The results from untargeted metabolomics of the follicular fluid revealed that MT supplementation was associated with an altered follicular fluid microenvironment characterized by the upregulation of estrogen derivatives and glutathione-related pathways. Transcriptomic analysis of blastocysts showed significant upregulation of the rate-limiting genes steroidogenic acute regulatory protein (STAR) and 3-hydroxy-3-methylglutaryl-CoA reductase (HMGCR), suggesting a potential role in promoting the synthesis of reproductive hormones and maintaining the progress of embryo development. The in vitro study showed that MT supplementation at a concentration of 10^−7^ mol/L in oocyte maturation media significantly improved cleavage rates, blastocyst rates, and total blastocyst cell numbers compared to the control. In vitro evaluations demonstrated that MT scavenged intracellular reactive oxygen species (ROS), while transcriptomic analysis indicated a reprogramming of the embryonic transcriptome. These transcriptomic changes were associated with the AMPK, FoxO, and autophagy pathways, as well as the modulation of endoplasmic reticulum stress and cellular senescence. The results from both the in vivo and in vitro studies suggested that MT supplementation was associated with a follicular microenvironment favorable for oocyte growth and protected embryos from oxidative damage. Therefore, MT supplementation effectively reduced the disrupted oocyte development caused by OPU and improved oocyte recovery and quality in general. These findings provide promising experimental evidence for the potential application of MT in dairy breeding and offer insights into the associated molecular pathways. However, further functional validation is required to fully elucidate the underlying mechanisms and establish its large-scale applicability.

## 1. Introduction

Assisted reproductive technologies (ARTs), including ovum pick-up (OPU) combined with in vitro embryo production (IVEP), sex-sorted semen, and embryo transfer are pivotal processes in modern cattle breeding. These technologies help to improve genetic selection and accelerate generation turnover [1,2]. In addition, they also contribute to minimizing the environmental footprint of dairy operations by boosting individual productivity. OPU is an essential procedure that enables high-frequency oocyte collection from the selected dairy cow to collect an average of 15 to 25 oocytes per session and potentially to generate over 50 offspring per donor annually [3,4]. OPU can also be successfully applied to prepubertal heifers, pregnant cows, and cows with uterine pathologies, accelerating the genetic advancement beyond the limitations of conventional multiple ovulation and embryo transfer (MOET) [3,4]. Consequently, the global application of OPU-IVEP has been significantly adopted, with the OPU-in vitro-produced embryo numbers outpacing in vivo-derived embryos nearly fourfold [5,6].

However, the rapidly applied implementation of OPU-IVEP has shortcomings regarding the compromised developmental competence of retrieved oocytes, and this constrains its optimal efficiency [7,8]. This is because both the quality and the quantity of the OPU oocytes are impacted by this process. Additionally, in vitro-matured oocytes suffer from oxidative stress, mitochondrial dysfunction, and incomplete cytoplasmic maturation, resulting in pregnancy rates 10–40% lower than those achieved with in vivo-derived embryos [7,8]. To address these constraints, donor metabolic management, follicle synchronization protocols, and targeted antioxidant strategies have been employed to fully capitalize on OPU technologies for dairy cattle breeding. Even though these procedures effectively circumvent some of the physiological limitations of standard MOET regimens, the overall outcomes of OPU-IVEP remain dependent on the quality and yield of retrieved cumulus–oocyte complexes (COCs) [9,10]. A tightly regulated follicular microenvironment is essential for oocytes to acquire full developmental competence [11], and the mechanical follicle aspiration combined with frequently exogenous gonadotropin stimulation occurring in OPU will inevitably compromise ovarian homeostasis [12,13]. These mechanical and endocrine stresses trigger systemic inflammation, leading to local inflammatory responses, redox imbalances, and metabolic dysregulation within the follicular fluid. These perturbations induce granulosa cell apoptosis and accelerated follicular atresia, ultimately undermining both oocyte retrieval yield and developmental competence. It has been reported that excessive intracellular reactive oxygen species (ROS) accumulation disrupts mitochondrial structural integrity and induces DNA double-strand breaks in oocytes [14,15]. Concurrently, immune cytokine dynamics shift toward a pro-inflammatory profile during post-OPU in oocytes [16]. Since the follicular fluid serves as the immediate microenvironmental niche supplying nutrients, energy substrates, and signaling molecules, the metabolic dysregulation, particularly in steroidogenesis and redox maintenance pathways, directly impairs oocyte maturation [17]. Therefore, strengthening endogenous antioxidant capacity, dampening inflammatory signaling, and optimizing follicular metabolic profiles represent promising avenues for improving OPU-IVEP efficacy [18,19].

MT is a polytropic molecule that regulates redox homeostasis, immune responses, circadian rhythms, and cellular metabolism [20,21]. It has been well-documented that in the female reproductive system of mammals, MT acts as a direct free-radical scavenger and also concurrently upregulates the endogenous antioxidant enzymes to mitigate oxidative damage [22,23], and this has been observed within the ovarian environment [24]. MT also orchestrates the balance between pro- and anti-inflammatory cytokines, shielding the follicular niche and the systemic circulation from immune disruption [25,26] and reprograms cellular energy utilization and lipid synthesis to maintain a nutrient-rich follicular fluid that supports oocyte competence [27]. In vitro studies have demonstrated that MT supplementation during oocyte maturation enhances nuclear and cytoplasmic maturation and the subsequent blastocyst formation rate in cattle. These effects are mediated via MT1 and MT2 G-protein coupled receptors, which trigger the expression of key genes (e.g., *HAS2*) governing cumulus expansion and antioxidant defense [28]. Furthermore, the in vivo studies with subcutaneous MT implants (40–80 mg) in ruminants show enhanced superovulatory responsiveness, higher corpora lutea counts, and greater numbers of viable embryos, culminating in improved pregnancy success [29]. Despite these promising findings, the effects of MT on OPU have not been fully investigated. Therefore, it remains largely unknown whether MT can alleviate the combined oxidative, endocrine, and metabolic stresses caused by routine OPU procedures in Holstein cows.

In this study, we aim to systematically explore the potential effects of MT on routine OPU procedures in Holstein cows. To achieve this goal, we have constructed protocols to measure the potential interactions between MT administration and exogenous follicle-stimulating hormone (FSH) stimulation during in vivo superstimulation and an integrated assessment linking in vivo follicular fluid metabolomics with in vitro embryo transcriptomics. The results show that subcutaneous administration of 80 mg MT per injection (cumulative dose: 320 mg) markedly improves in vivo oocyte quality. These improvements were associated with reduced circulating progesterone levels and alterations in the follicular fluid metabolic microenvironment, correlating with the upregulation of glutathione-related antioxidant pathways and increased STAR and HMGCR expression. Additionally, in vitro supplementation with 10^−7^ mol/L MT enhances early embryonic development and reduces intracellular ROS levels. Concurrently, transcriptomic reprogramming indicates a potential involvement of AMPK/FoxO-related pathways and suggests a downregulation of endoplasmic reticulum (ER) stress signatures. Overall, the in vitro and in vivo studies of MT supplementation offer a practical approach to maximize OPU efficiency in dairy cattle breeding.

## 2. Materials and Methods

### 2.1. Animals

*In vivo*, this study was conducted at the farm of the Inner Mongolia Saikexing Institute of Breeding and Reproductive Biotechnology in Domestic Animals, Hohhot, Inner Mongolia Autonomous Region, China (40.515396° N, 111.843311° E). Healthy, nulliparous Holstein heifers at approximately 13 months of age with a similar body condition score (BCS = 3.0) were randomly selected as donors. In vitro, the ovaries were collected from a local slaughterhouse. All experimental cows were raised and managed under identical environmental conditions, and all animal experiments were approved by the Animal Ethics Committee of China Agricultural University (Approval Code: AW01806202-1-11 on 5 June 2024).

### 2.2. Experimental Design

Criteria for excluding animals or data points were not established a priori. Forty healthy nulliparous Holstein heifers (approximately 13 months of age) with good reproductive function and high breeding value were selected. To ensure an equal distribution of age, a stratified randomization approach was applied to allocate the animals into four groups (one control group and three MT treatment groups).

**Figure 1:**
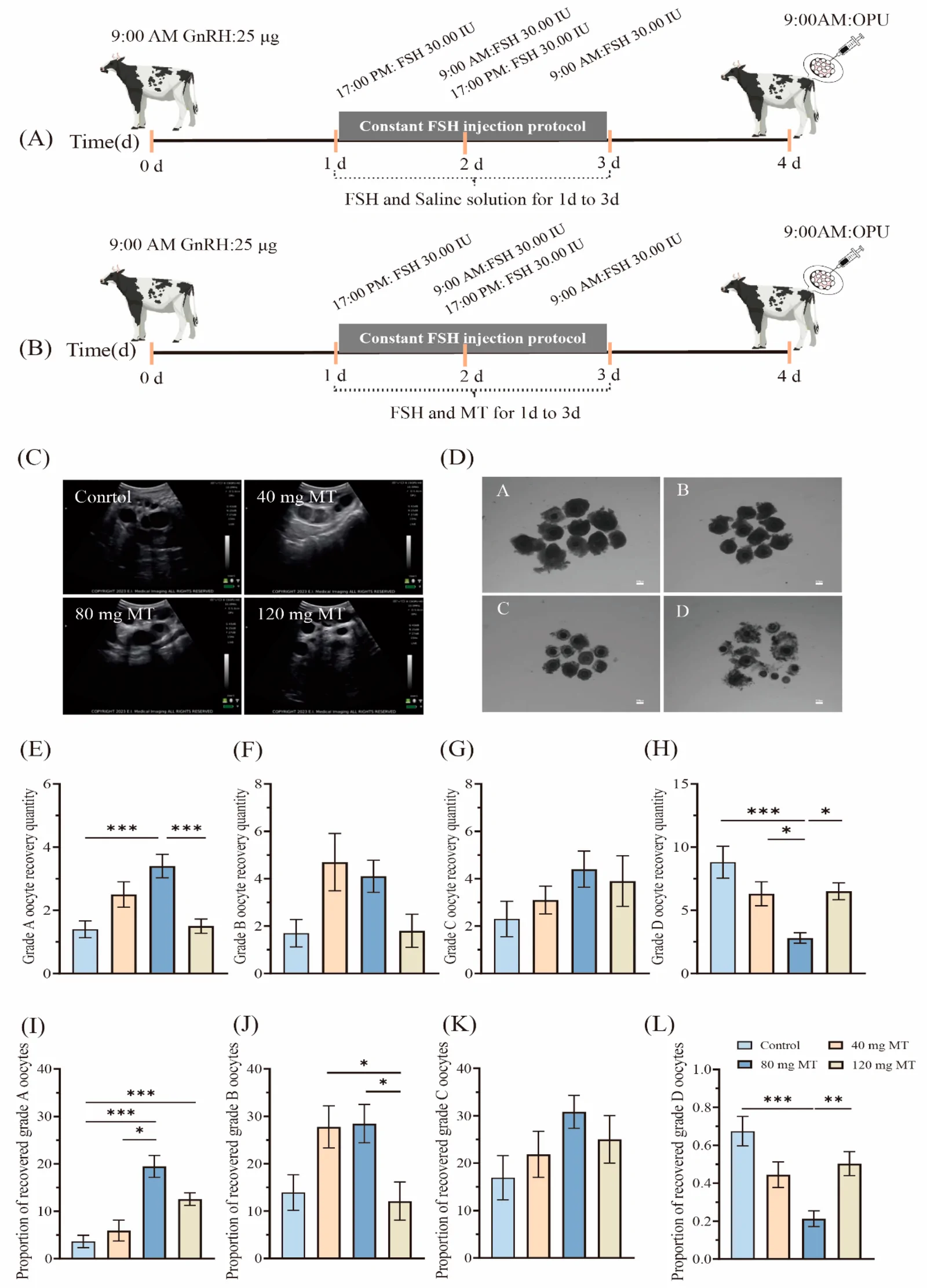
Effects of melatonin subcutaneous injection on the number and quality of the oocytes in dairy cows. (**A**) Superovulation protocol for the control group. (**B**) Superovulation with melatonin injection protocol. (**C**) B-mode ultrasound image during OPU. (**D**) Morphological classification of oocytes in grades. (**E**–**H**) Number of recovered Grade A (**E**), Grade B (**F**), Grade C (**G**), and Grade D (**H**) oocytes. (**I**–**L**) Recovery rates of Grade A (**I**), Grade B (**J**), Grade C (**K**), and Grade D (**L**) oocytes. *n* = 10 for each group. * *p* < 0.05, ** *p* < 0.01, *** *p* < 0.001.

The treatment regimens were as follows: The day of GnRH (Ningbo Sansheng Biological Technology, Ningbo, China) injection (25 μg/head) was designated as day 0. FSH (Ningbo Second Hormone Factory, Ningbo, China) was injected intramuscularly according to the following schedule: at 5:00 p.m. on day 1; at 9:00 a.m. and 5:00 p.m. on day 2; and at 9:00 a.m. on day 3. Concurrently with each of the four FSH injections, cows in the MT treatment groups were subcutaneously injected with 40, 80, or 120 mg MT (Sigma-Aldrich Corporation, St. Louis, MO, USA) per administration. Consequently, over the course of the superstimulation protocol, the cumulative MT dose received by each animal in the respective treatment groups was 160, 320, or 480 mg. The control group received an equal volume of normal saline at each time point (Figure 1B).

OPU was performed on day 4. Blood samples were collected prior to GnRH injection on day 0, prior to FSH injection on day 3, and 1 h after the completion of the OPU procedure on day 4 (which corresponded to 24–26 h after the final exogenous FSH injection) for the measurement of inflammatory factors, oxidative stress indicators, and reproductive hormones. Follicular fluid was also collected during the OPU procedure on day 4 for metabolomic analysis. All experimental animals were housed under identical environmental conditions and subjected to standard farm management practices.

### 2.3. Ovum Pick-Up (OPU) Procedure

The cows were anesthetized with 5 mL of 5% lidocaine hydrochloride (Harbin Sanma Veterinary Medicine Co., Ltd., Harbin, China). After cleaning the feces, the ovaries were stabilized by rectal palpation. Follicles with 2–8 mm in diameter were punctured under ultrasound guidance (IBEX Pro; E.I Medical Imaging, Zhengzhou, China), and the follicular fluid was collected into a preheated 50 mL centrifuge tube. After transport to the laboratory, the follicular fluid was washed and filtered repeatedly through an oocyte collection cup. Once the fluid became clear, oocytes were identified and collected under a microscope. Oocyte quality was classified into four grades (A, B, C, and D) according to morphological characteristics.

### 2.4. Collection and Culture of Oocytes

The healthy bovine ovaries collected from a slaughterhouse in Hohhot City were washed and stored in preheated normal saline containing gentamicin (Ruicheng Lvman Biopharmaceutical Co., Ltd., Ruicheng, China) and transported to the laboratory within 4 h. The tissue surrounding the ovaries was removed, and follicular fluid was aspirated from follicles with 2–8 mm diameters by a 20 mL syringe. Cumulus–oocyte complexes (COCs) with uniform cytoplasm and intact surrounding granulosa cell layers were selected under a stereomicroscope (SMZ745T; Nikon, Tokyo, Japan). The COCs were washed three times with the oocyte manipulation medium. For each experimental run, the collected COCs were pooled and randomly allocated into four groups. They were then transferred into BO-IVM medium (Huayuehang Instrument Co., Ltd., Guangzhou, China, Cat. No.: 71001) and treated with MT at concentrations of 0 (control), 10^−4^, 10^−7^, and 10^−10^ mol/L. All four treatments were performed concurrently within each run. The COCs were cultured in a cell incubator (NUAIR-NU-5510E; NuAire, Plymouth, MN, USA) at 38.5 °C with 5% CO_2_ and saturated humidity for 22 h, with 50 COCs cultured per 500 μL of medium. One independent biological replicate was defined as a distinct batch of COCs collected and processed on a single day. The entire in vitro experiment was repeated for a total of four independent biological replicates.

### 2.5. In Vitro Fertilization (IVF)

Fertilization droplets of 90 μL BO-IVF (Cat. No.: 71004) were prepared in a 60 mm dish and equilibrated for 4 h at 38.5 °C. Mature oocytes were transferred to oocyte manipulation medium, washed three times, and then further washed three times with the fertilization droplets before fertilization. The frozen semen was removed from the liquid nitrogen tank and thawed in a 38 °C water bath for 1 min. Then, the semen was transferred to a centrifuge tube containing semen washing solution (Huayuehang Instrument Co., Ltd., Guangzhou, China, Cat. No.: 71003) for centrifugation at 1500 r/min for 5 min. After two centrifugation cycles, the sperm suspension was slowly added to the fertilization droplets containing the oocytes, and the final sperm concentration was adjusted to 1 × 10^6^ cells/mL. The mixture was incubated at 38.5 °C with 5% CO_2_ and saturated humidity for 18–22 h.

### 2.6. In Vitro Embryo Culture (IVC)

First, the zygotes were gently pipetted to remove the surrounding cumulus cells and then washed three times with preheated development medium (Huayuehang Instrument Co., Ltd., Guangzhou, China, Cat. No.: 71005). After 22–24 h of in vitro fertilization, the treated zygotes were transferred to pre-equilibrated development medium microdrops and cultured in a tri-gas incubator (K-MINC-1000; Cook Medical, Bloomington, IN, USA) with 5% O_2_, 5% CO_2_, and saturated humidity. The day of fertilization was designated as day 0. Cleavage was assessed after 48 h of culture, and the number of blastocysts was recorded at 168 h.

### 2.7. ROS and DAPI Staining

To investigate whether MT supplementation affects ROS levels in oocytes, these levels were first assessed. 2′,7′-dichlorofluorescin diacetate (DCFH-DA) is a fluorogenic dye used to measure ROS activity. DCFH-DA itself is non-fluorescent and can freely cross the cell membrane. After cellular uptake, DCFH-DA is deacetylated by cellular esterases to a non-fluorescent compound, which is later oxidized by ROS into 2′,7′-dichlorofluorescein (DCF). DCF is a fluorescent compound with maximum excitation and emission wavelengths of 495 nm and 529 nm, respectively. Samples were fixed with 4% paraformaldehyde for 15 min and then washed with DPBS. Subsequently, the samples were stained with DAPI (1 μg/mL) for 10 min at room temperature in the dark. After washing, the samples were observed and photographed under a fluorescence microscope (FLUOVIEW FV1000; Olympus Corporation, Tokyo, Japan). To ensure accurate quantitative comparisons, all experiments were performed with at least three independent biological replicates (defined as independent experimental runs conducted on different days using distinct batches of oocytes or embryos). For ROS quantification in oocytes, approximately 10 oocytes were analyzed per replicate. For blastocyst evaluation, including cell counting based on DAPI-positive nuclei and ROS assessment, 3 blastocysts were randomly selected and imaged per biological replicate. Image exposure parameters were kept strictly constant across all comparative groups, and the total number of cells was counted using ImageJ (version 2.14.0; NIH, Bethesda, MD, USA).

### 2.8. Detection of Antioxidants, Immune Factors, and Related Indicators in Serum

Serum concentrations of follicle-stimulating hormone (FSH), luteinizing hormone (LH), progesterone (P4), and estradiol (E2) were quantified using enzyme-linked immunosorbent assay (ELISA) kits (Nanjing Jiancheng Bioengineering Institute, Nanjing, China). The assays were performed by Beijing Jinhaikeyu Biological Technology Development Co., Ltd. (Beijing, China). The specific catalog numbers are as follows: FSH (Cat: JH00159), LH (Cat: JH00160), P4 (Cat: JH00163), and E2 (Cat: JH00164). The analytical sensitivities of the assays were <0.1 mIU/mL for FSH, <0.1 mIU/mL for LH, <0.1 ng/mL for P4, and <0.1 pg/mL for E2. The intra-assay and inter-assay coefficients of variation (CV) were less than 10% and 15%, respectively. Furthermore, to minimize potential interference of the exogenous FSH on the quantification of endogenous FSH, blood collection on day 4 was performed 24–26 h after the final exogenous FSH injection.

### 2.9. Metabolomics Analysis

On day 4, follicular fluid was collected during OPU from Holstein cows in the 80 mg/injection MT treatment group (*n* = 6) and the control group (*n* = 6); the fluid was centrifuged for 10 min. The resulting supernatant was initially frozen at −20 °C and subsequently transferred to liquid nitrogen for storage prior to metabolomic analysis. Untargeted metabolomics analysis was performed using an ultra-high-performance liquid chromatography Fourier transform mass spectrometry (UHPLC-FTMS) system. The detailed procedures were as follows:

(1) Sample preparation: A 100 μL aliquot of each sample was mixed with 400 μL of pre-cooled extraction solvent (acetonitrile: methanol = 1:1, *v*/*v*) containing 0.02 mg/mL L-2-chlorophenylalanine as an internal standard. The mixture was vortexed for 30 s and sonicated at low temperature (5 °C, 40 kHz) for 30 min. Following incubation at −20 °C for 30 min, the samples were centrifuged at 12,000 rpm for 15 min at 4 °C. The supernatant was collected, evaporated under a gentle stream of nitrogen, and the residue was reconstituted in 100 μL of solvent (acetonitrile: water = 1:1, *v*/*v*). After a second sonication (5 °C, 40 kHz, 5 min) and centrifugation at 12,000 rpm (4 °C, 10 min), the final supernatant was transferred to an autosampler vial equipped with a glass insert for LC-MS/MS analysis.

(2) Quality control (QC): QC samples were prepared by pooling equal volumes of all experimental samples. To monitor system stability and analytical reproducibility, one QC sample was injected after every 5 to 15 experimental samples throughout the analytical run.

(3) LC-MS/MS analysis: Metabolomic profiling was conducted using a Thermo Scientific UHPLC-Q Exactive HF-X mass spectrometry system (Majorbio Bio-Pharm Technology Co., Ltd., Shanghai, China).

(4) Chromatographic conditions: A 3 μL aliquot of the sample was injected onto an HSS T3 column (100 mm × 2.1 mm i.d., 1.8 µm) maintained at 40 °C, with a flow rate of 0.40 mL/min. The mobile phase consisted of solvent A (95% water and 5% acetonitrile, containing 0.1% formic acid) and solvent B (47.5% acetonitrile, 47.5% isopropanol, and 5% water, containing 0.1% formic acid).

(5) Mass spectrometry conditions: Data acquisition was performed in both positive (ESI+) and negative (ESI-) electrospray ionization modes with a mass range of 70–1050 m/z. The operational parameters were set as follows: sheath gas flow rate, 50 psi; auxiliary gas flow rate, 13 psi; auxiliary gas heater temperature, 425 °C; ion spray voltage, 3500 V (positive mode) and −3500 V (negative mode); ion transfer tube temperature, 325 °C; and normalized collision energy (NCE), 20, 40, and 60 eV.

(6) Data analyses: No animals or biological samples were excluded from the analysis. For the metabolomic analyses, standard quality control filtering was applied to remove low-quality features, but no entire sequencing libraries or sample replicates were excluded. All data analyses were performed using R software (version 4.2.2) and MetaboAnalyst 5.0. Unsupervised principal component analysis (PCA) was initially conducted to visualize the overall separation between the MT treatment and control groups and to screen for potential outliers (defined as samples outside the 95% confidence ellipse). No samples fell outside this ellipse; therefore, all samples were retained for subsequent downstream analyses**.** Supervised orthogonal partial least squares discriminant analysis (OPLS-DA) was then applied to maximize metabolic discrimination between the groups. The robustness of the OPLS-DA model was validated via a 200-iteration permutation test, with R^2^Y > 0.5 and Q^2^ > 0.4 indicating a valid model. Differentially abundant metabolites (DAMs) were identified using a combination of multivariate and univariate criteria: variable importance in projection (VIP) > 1 from the OPLS-DA model, *p* < 0.05 from Student’s *t*-test (based on normalized peak areas and adjusted using the Benjamini–Hochberg false discovery rate method), and |fold change (FC)| > 1.2. Functional enrichment analysis of the DAMs was performed using the KEGG database to identify significantly perturbed metabolic pathways (Fisher’s exact test, FDR-adjusted *p* < 0.05). Furthermore, Pearson correlation analysis was conducted to evaluate the linear relationships between key DAMs and oocyte quality parameters, with statistical significance defined as *p* < 0.05. The differentially abundant metabolites, identified using FDR-adjusted *p* < 0.05 alongside VIP and fold-change thresholds, are listed in Table S1.

### 2.10. Blastocyst Transcriptomic Sequencing

Blastocysts from the MT-treated ($10^{-7}$ mol/L) (*n* = 3) and control (*n* = 3) groups were snap-frozen in liquid nitrogen and stored at −80 °C until RNA isolation. For each group, the three biological replicates were obtained from three independent in vitro production (IVP) runs. Each biological replicate consisted of a pool of 3 blastocysts. The transcriptomes of the blastocysts were evaluated using the Smart-seq2 protocol [30]. Briefly, total RNA was extracted, and libraries were constructed and sequenced on an Illumina NovaSeq 6000 platform.

For data processing, fastp (v0.20.1) and FastQC (v0.11.9) were used for data quality control [31]. The principal RNA-seq quality-control metrics for each biological replicate, including sequencing depth, proportion of retained clean reads, and mapping rate, are detailed in Supplementary Table S2. Clean reads were aligned to the bovine reference genome (Bos_taurus.ARS-UCD1.3) with the corresponding genome annotation (Bos_taurus.ARS-UCD1.3) using STAR 2.7.9a software [32]. Gene counting was performed with featureCounts (Subread 1.6.3) [33], and DESeq2 [34] was used to identify differentially expressed genes. Genes showing significant differences (FDR-adjusted *p* < 0.05) were subjected to GO enrichment analysis with g:Profiler [35]. Additionally, Kyoto Encyclopedia of Genes and Genomes (KEGG) pathway enrichment analysis was performed using g:Profiler. The Bos taurus genome was used as the background gene set. The statistical significance of enrichment was determined using a hypergeometric test, and pathways with an FDR-adjusted *p*-value < 0.05 (after multiple-testing correction) were considered significantly enriched. No embryos were excluded from the analysis. For the transcriptomic analyses, standard quality control filtering was applied to remove low-quality features, but no entire sequencing libraries or sample replicates were excluded. The differentially expressed genes are listed in Table S3.

### 2.11. Statistical Analysis

All animals were included in the final analysis. Unless otherwise specified, for endpoints measured only at a single time point, a standard one-way ANOVA was utilized, followed by Tukey’s post hoc test. Data are presented as mean ± standard error of the mean (SEM). A *p*-value < 0.05 was considered statistically significant. Data were analyzed using GraphPad Prism (version 8.0.2, GraphPad Software, La Jolla, CA, USA). Because the experimental design involved repeated measurements on the same animals over time, data were analyzed using a Mixed-Effects Model rather than treating repeated measurements as independent observations. The individual cow was considered the experimental unit. In the model, melatonin treatment dose, time, and the treatment × time interaction were entered as fixed effects, while cow identification number was included as a random effect to account for within-subject correlation. The assumption of normality was verified by inspecting the Q-Q plots of the model residuals. Where a significant main effect or interaction was detected, post hoc pairwise comparisons were performed using Tukey’s multiple comparisons test to adjust for multiple comparisons. For the in vitro embryo development experiments, the experimental unit was defined as an independent in vitro production (IVP) run. Oocytes collected within a single run were pooled and randomly allocated to the respective treatment groups. Developmental outcomes (cleavage and blastocyst rates) were calculated per independent run, and statistical analyses were performed at the replicate level, rather than at the individual oocyte/embryo level. Similarly, to avoid pseudoreplication in the downstream assays evaluating oxidative stress and blastocyst quality, the independent IVP run was maintained as the experimental unit. Data from individual oocytes or blastocysts sampled within the same run were averaged to generate a single run-level mean. Statistical analyses for these parameters were then performed using these run-level means (*n* = 4 independent runs).

## 3. Results

### 3.1. Effects of MT Supplementation on Superovulation Performance and Oocyte Quality in Holstein Cows

In the in vivo study, Holstein cows were subjected to a standard FSH superovulation protocol combined with subcutaneous MT supplementation at doses of 0, 40, 80, and 120 mg before OPU (Figure 1A,B). Representative ultrasonography was performed to monitor follicular development during the OPU procedure (Figure 1C). After OPU, the morphological grading of the recovered cumulus-oocyte complexes (COCs) showed markedly improved overall oocyte quality in the 80 mg/injection MT-treated group, which yielded the highest total count and percentage of high-quality Grade A oocytes compared to the control and 120 mg/injection MT groups (*p* < 0.001) (Figure 1E,I). Conversely, both the number and proportion of poor-quality Grade D oocytes were significantly reduced in the 80 mg/injection MT-treated group compared to the control (*p* < 0.001) (Figure 1H,L). Although the absolute counts of Grade B and C oocytes did not differ among groups (Figure 1F,G), the proportions of Grade B oocytes were significantly elevated in the 40 and 80 mg/injection MT groups compared to the control group (*p* < 0.05) (Figure 1J). These results indicated that 80 mg MT per administration (cumulative dose: 320 mg) was the most effective dose among those evaluated for enhancing the morphological quality and yield of in vivo recovered COCs; therefore, further investigations of the follicular microenvironment were conducted using this dose.

### 3.2. Effects of MT on the Metabolic Profile of Bovine Follicular Fluid

**Figure 2:**
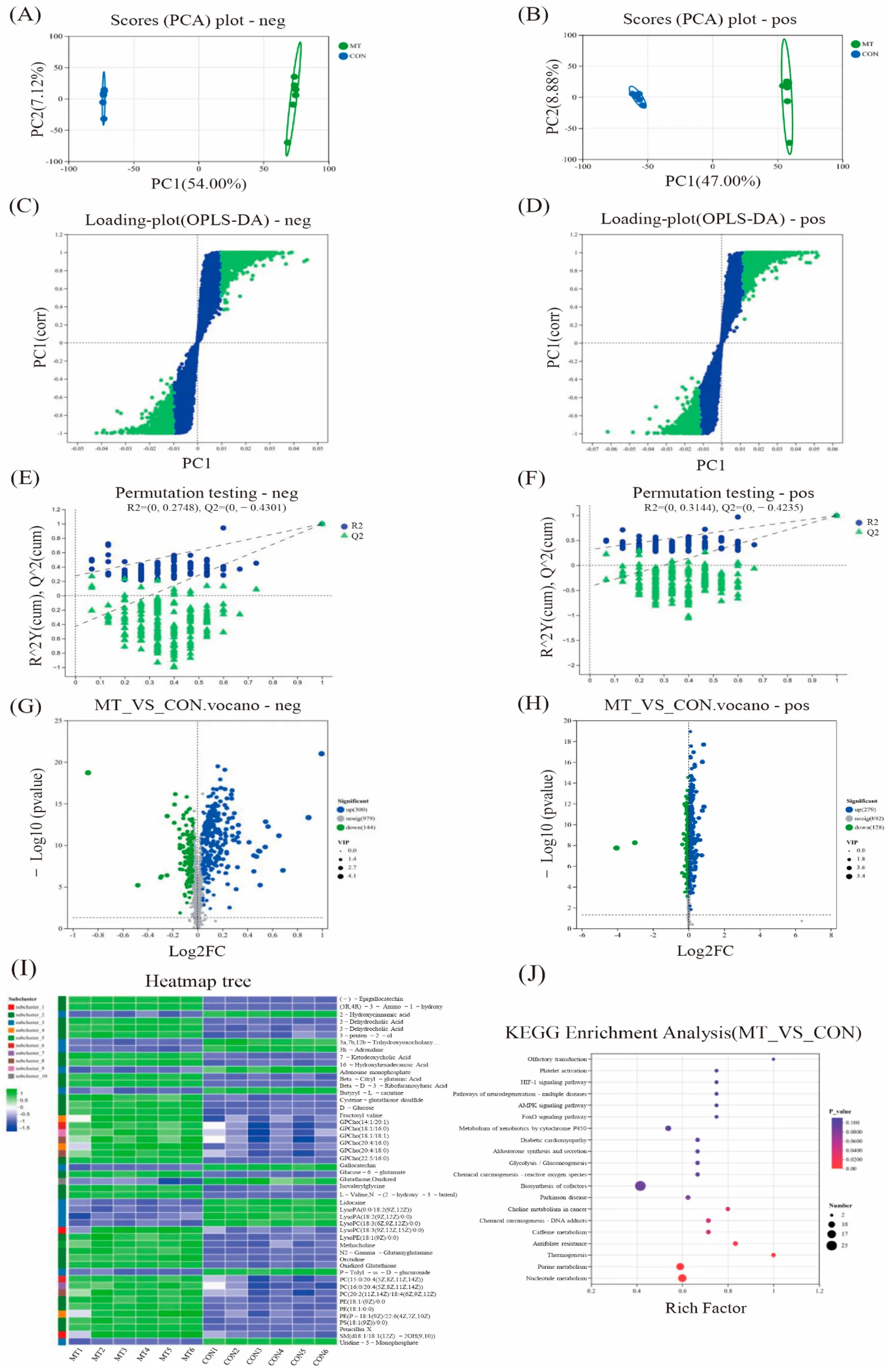
Effects of subcutaneous injection of melatonin on the untargeted metabolomics of follicular fluid of dairy cows. (**A**,**B**): Principal component analysis (PCA) score plots in negative (**A**) and positive (**B**) ion modes. (**C**,**D**): Orthogonal partial least squares discriminant analysis (OPLS-DA) score plots in negative and positive (**D**) ion modes. (**E**,**F**): Validation plots of the OPLS-DA models using a 200-iteration permutation test in negative (**E**) and positive (**F**) ion modes. (**G**,**H**): Volcano plots of differential metabolites in negative (**G**) and positive (**H**) ion modes. (**I**): Hierarchical clustering heatmap of differential metabolites. (**J**): Kyoto Encyclopedia of Genes and Genomes (KEGG) pathway enrichment analysis of differential metabolites. (*n* = 6 for each group).

To identify the effects of MT on the follicular fluid metabolome, untargeted metabolomics was performed. Principal component analysis (PCA) in both negative (Figure 2A) and positive (Figure 2B) ion modes revealed a distinct separation between the MT and control (CON) groups, indicating a fundamental shift in the global metabolic profile following MT supplementation. To maximize the metabolic discrimination between the MT treatment and control groups, supervised orthogonal partial least squares discriminant analysis (OPLS-DA) was applied. The OPLS-DA score plots (Figure 2C,D) clearly confirmed a distinct separation between the two groups in both ionization modes. Furthermore, the robustness of the OPLS-DA models was validated using a 200-iteration permutation test. For the positive ionization mode, the model exhibited strong explanatory and predictive capabilities with R^2^Y = 0.998 and Q^2^ = 0.994. The permutation test confirmed the model was not overfitted, as indicated by the y-intercepts (R^2^ intercept = 0.3144, Q^2^ intercept = −0.4235). Similarly, for the negative ionization mode, the model yielded R^2^Y = 0.999 and Q^2^ = 0.997, with the permutation test also indicating a valid model without overfitting (R^2^ intercept = 0.2748, Q^2^ intercept = −0.4301) (Figure 2E,F). Volcano plots visualized the differentially abundant metabolites (DAMs), revealing 279 significantly upregulated and 128 downregulated metabolites in the positive ion mode, as well as 300 upregulated and 144 downregulated metabolites in the negative ion mode in the MT group compared to the control group (Figure 2G,H). The hierarchical clustering of the top DAMs showed highly consistent intra-group metabolic patterns and distinct inter-group variations (Figure 2I). KEGG pathway enrichment analysis showed that these DAMs were significantly enriched in critical metabolic pathways, including nucleotide metabolism, purine metabolism, thermogenesis, and the AMPK signaling pathway (Figure 2J). Thus, MT appears to optimize the follicular microenvironment primarily by modulating energy homeostasis and nucleotide biosynthesis, which we examined in detail in the following studies.

### 3.3. Effects of MT on Glutathione Metabolism and Systemic Oxidative Stress

**Figure 3:**
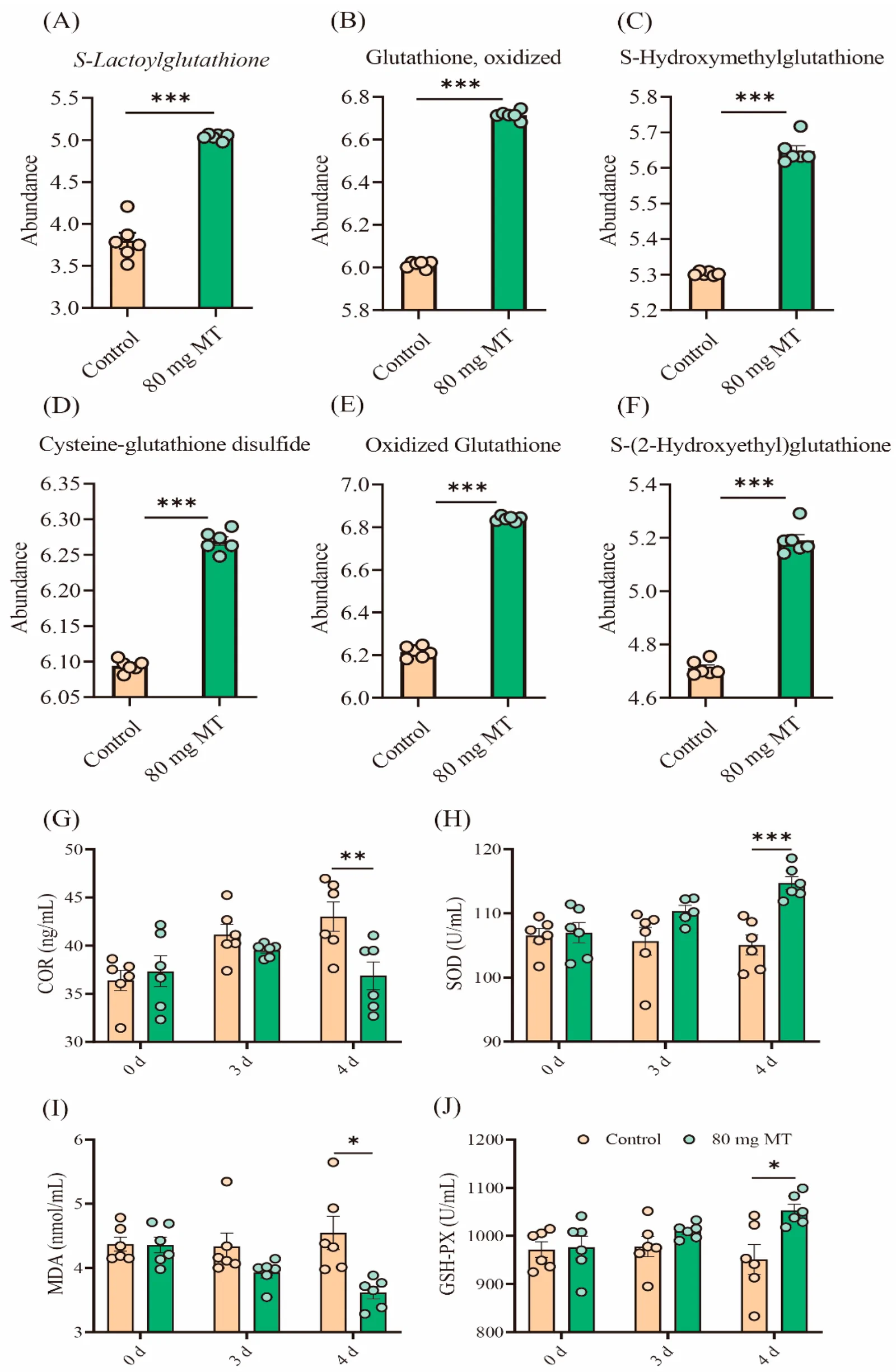
Effects of melatonin subcutaneous injection on glutathione metabolism, cortisol level, SOD activity and lipid pro-oxidation in dairy cows. (**A**–**F**) Relative abundance of glutathione-related differential metabolites in follicular fluid, including S-Lactoylglutathione (**A**), Glutathione, oxidized (**B**), S-hydroxymethylglutathione (**C**), Cysteine-glutathione disulfide (**D**), Oxidized Glutathione (**E**), and S-(2-Hydroxyethyl) glutathione (**F**). (**G**–**J**) Serum levels of cortisol (COR, (**G**)), Superoxide dismutase (SOD, (**H**)), Malondialdehyde (MDA, (**I**)), and Glutathione peroxidase (GSH-Px, (**J**)) at days 0, 3, and 4 of OPU. Orange boxes represent the control group, and green boxes represent the 80 mg/injection MT group. * *p* < 0.05, ** *p* < 0.01, *** *p* < 0.001. (*n* = 6 for each group).

For the redox-related pathways, the glutathione metabolism pathway was significantly upregulated in the follicular fluid of the 80 mg/injection MT group compared with the control. This included significant increases in the relative abundances of key glutathione derivatives, namely S-lactoylglutathione, oxidized glutathione, S-hydroxymethylglutathione, cysteine-glutathione disulfide, and S-(2-hydroxyethyl) glutathione (*p* < 0.001) (Figure 3A–F).

The results also showed that this local metabolic shift yielded systemic physiological benefits during superovulation and OPU. On day 4 (post-OPU), the serum concentrations of the stress hormone cortisol (COR) (*p* < 0.01) (Figure 3G) and the lipid peroxidation marker malondialdehyde (MDA) were significantly reduced (*p* < 0.05) (Figure 3I), while the activities of antioxidant enzymes including superoxide dismutase (SOD) (*p* < 0.001) and glutathione peroxidase (GSH-Px) (*p* < 0.05) were significantly increased in the MT group compared to the control group (Figure 3H,J). The results confirmed the protective effects of MT on OPU-induced physiological and oxidative stress.

### 3.4. Effects of MT on Follicular Steroidogenesis and Systemic Reproductive Hormone Profiles

**Figure 4:**
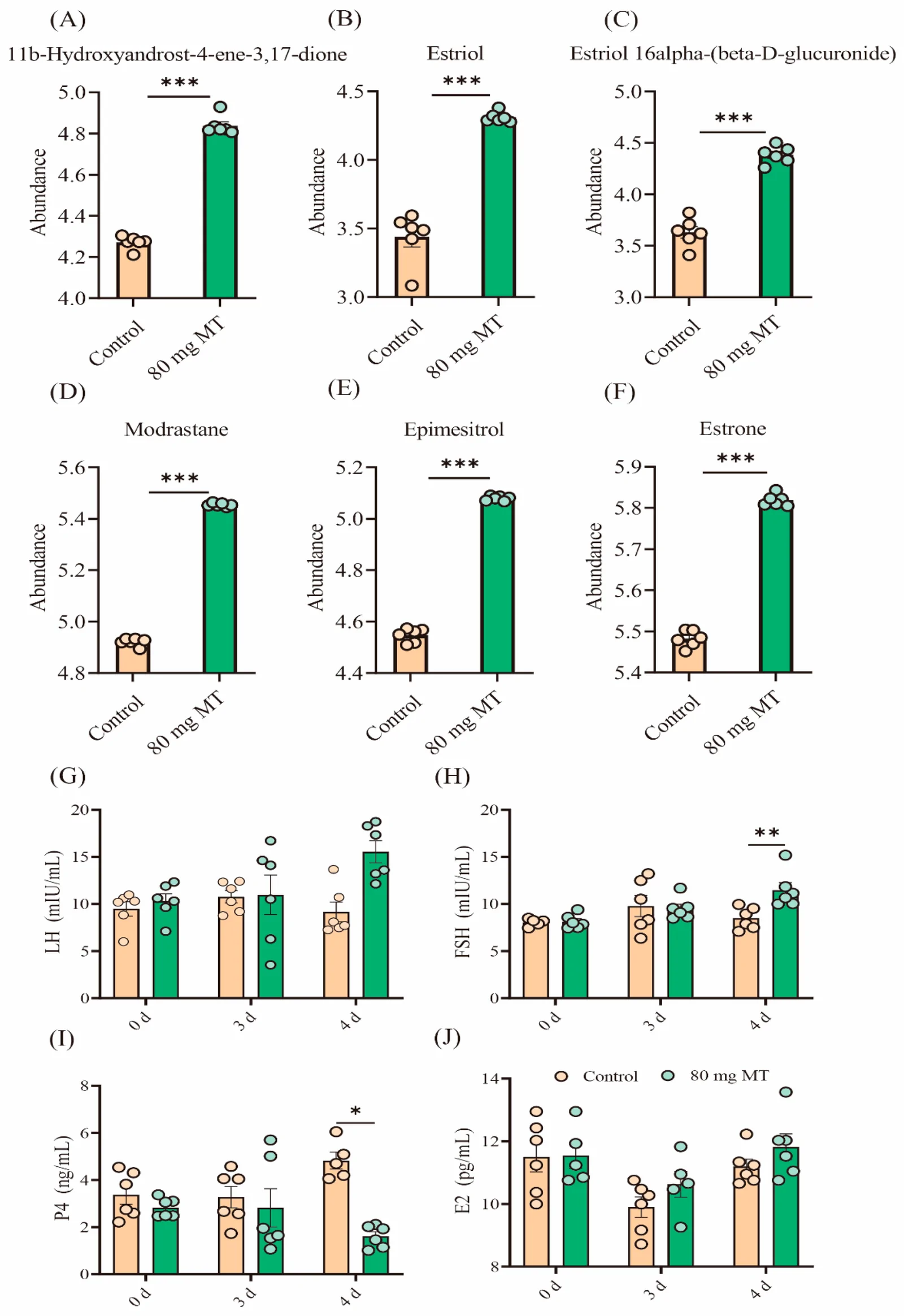
Effects of subcutaneous melatonin injection on steroid hormone metabolism and systemic reproductive hormone profiles in dairy cows. (**A**–**F**) Relative abundance of steroid and estrogen-related differential metabolites in follicular fluid, including 11b-Hydroxyandrost-4-ene-3,17-dione (**A**), Estriol (**B**), estriol 16α-(β-D-glucuronide) (**C**), Modrastane (**D**), Epimestrol (**E**), and Estrone (**F**). (**G**–**J**) Serum levels of reproductive hormones, including luteinizing hormone (LH, (**G**)), follicle-stimulating hormone (FSH, (**H**)), progesterone (P4, (**I**)), and estradiol (E2, (**J**)) at days 0, 3, and 4 of OPU. Orange boxes represent the control group, and green boxes represent the 80 mg MT per injection group. * *p* < 0.05, ** *p* < 0.01, *** *p* < 0.001. (Control, *n* = 6; 80 mg MT per injection group = 6).

The metabolomic analysis of the follicular fluid also revealed the beneficial effects of MT on steroidogenesis. The relative abundances of key steroid hormones and estrogen derivatives, including 11β-hydroxyandrost-4-ene-3,17-dione, estriol, estriol 16α-(β-D-glucuronide), modrastane, epimestrol, and estrone, were significantly increased in the 80 mg MT per injection (cumulative dose: 320 mg) compared with the control (*p* < 0.001) (Figure 4A–F). This enhanced estrogenic microenvironment within the follicles is essential for oocyte maturation.

The results showed that this local steroidogenic shift was accompanied by systemic endocrine alterations. On day 4 post-OPU, MT-treated cows exhibited significantly higher serum levels of follicle-stimulating hormone (FSH) (*p* < 0.01) (Figure 4H), and lower progesterone (P4) compared to the control group (*p* < 0.05) (Figure 4I). This high level of FSH provided sustained gonadotropic support for follicular development, and the lower serum P4 indicated an altered endocrine profile with reduced circulating progesterone following the OPU procedure. On day 4, the luteinizing hormone (LH) and estradiol (E2) levels were numerically higher in the MT group compared to the control group, although these differences did not reach statistical significance (Figure 4G,J). Collectively, these results demonstrated that MT optimizes both the local and systemic endocrine milieu to support superovulation.

### 3.5. Effects of MT Supplementation on In Vitro Maturation and Early Embryonic Development of Bovine Oocytes

**Figure 5:**
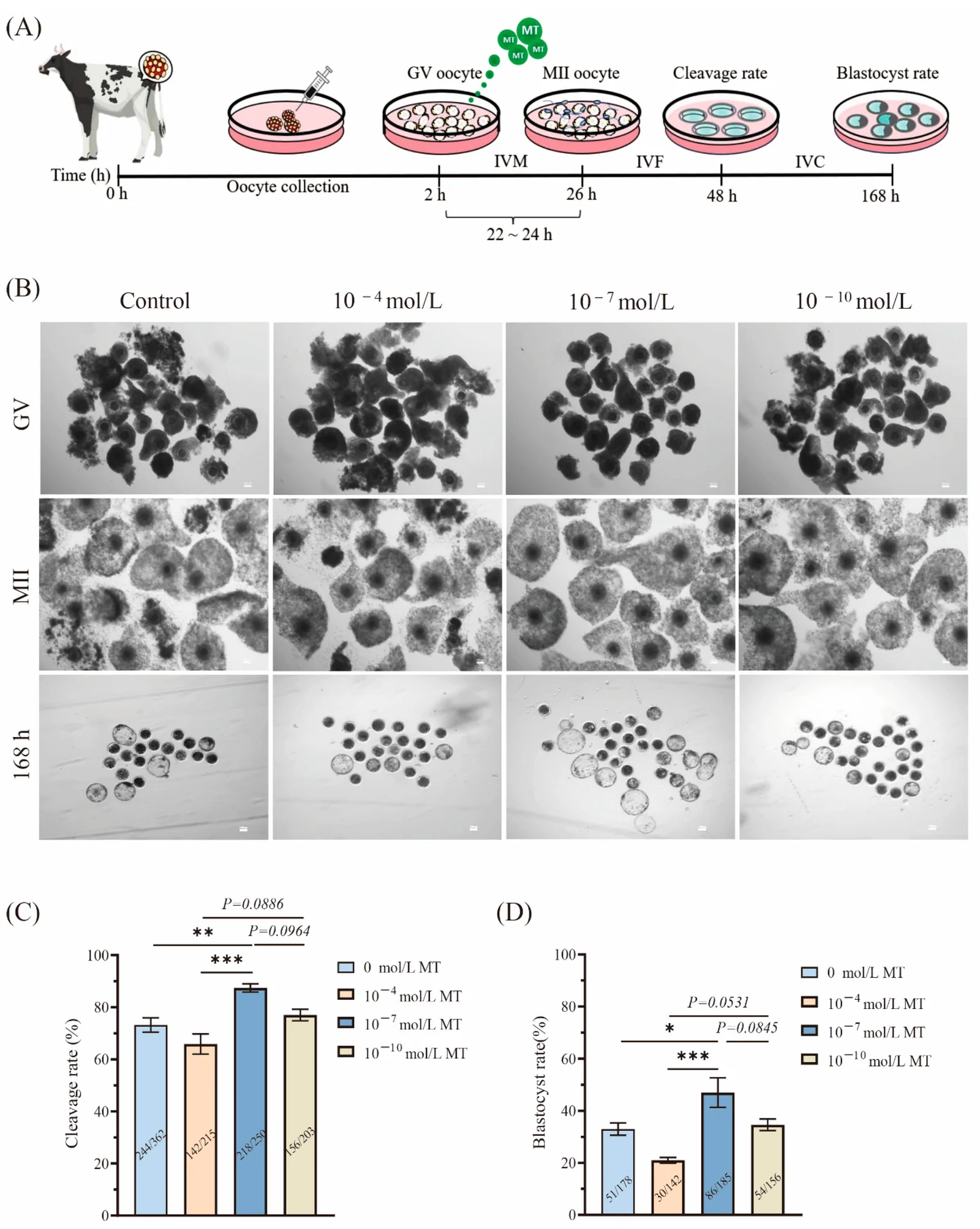
Effects of different melatonin concentrations in culture medium on the in vitro maturation and early embryonic development of bovine oocytes. (**A**). Schematic diagram of the in vitro culture process. (**B**). Representative morphological images of oocytes at the GV and MII stages, and embryos at 168 h of in vitro culture (blastocyst stage). Scale bars are 100 μm. (**C**,**D**). Cleavage (**C**) and blastocyst (**D**) rates of bovine embryos. The fractions inside the bars indicate the number of cleaved embryos/total cultured oocytes (**C**) and blastocysts/cultured zygotes (**D**). * *p* < 0.05, ** *p* < 0.01, *** *p* < 0.001. Exact *p*-values are shown for marginal significance. Data are presented as mean ± SEM from 4 independent biological replicates. A total of (control = 362, 10^−4^ mol/L MT = 215, 10^−7^ mol/L MT = 215, 10^−10^ mol/L MT = 203) oocytes were analyzed per group across all replicates. A total of (control = 178, 10^−4^ mol/L MT = 142, 10^−7^ mol/L MT = 185, 10^−10^ mol/L MT = 156) embryos were analyzed per group across all replicates.

To determine whether MT exerts a direct beneficial effect on oocyte quality independent of the maternal systemic environment, several concentrations of MT (0, 10^−4^, 10^−7^, and 10^−10^ mol/L) were added to the oocyte in vitro maturation (IVM) medium. The morphological evaluation at the germinal vesicle (GV), metaphase II (MII), and blastocyst (168 h) stages revealed that MT, particularly at 10^−7^ mol/L, significantly promoted cumulus cell expansion and blastocyst formation compared to the control (Figure 5A). Quantitative analysis confirmed these dose-dependent effects of melatonin on oocyte maturation. The cleavage rate (Figure 5C) and the blastocyst rate (Figure 5D) were significantly higher in the 10^−7^ mol/L group compared to the control (*p* < 0.05). Notably, the highest concentration (10^−4^ mol/L) appeared to have deleterious effects, yielding significantly lower developmental rates compared to the control or other melatonin-treated group (*p* < 0.01) (Figure 5C,D). These in vitro findings showed that 10^−7^ mol/L was the most effective concentration among those evaluated for directly enhancing bovine oocyte quality and subsequent embryonic development, prompting a closer look at the underlying cellular mechanisms.

### 3.6. Effects of MT on Oxidative Stress in MII Oocytes and Subsequent Blastocyst Quality In Vitro

**Figure 6:**
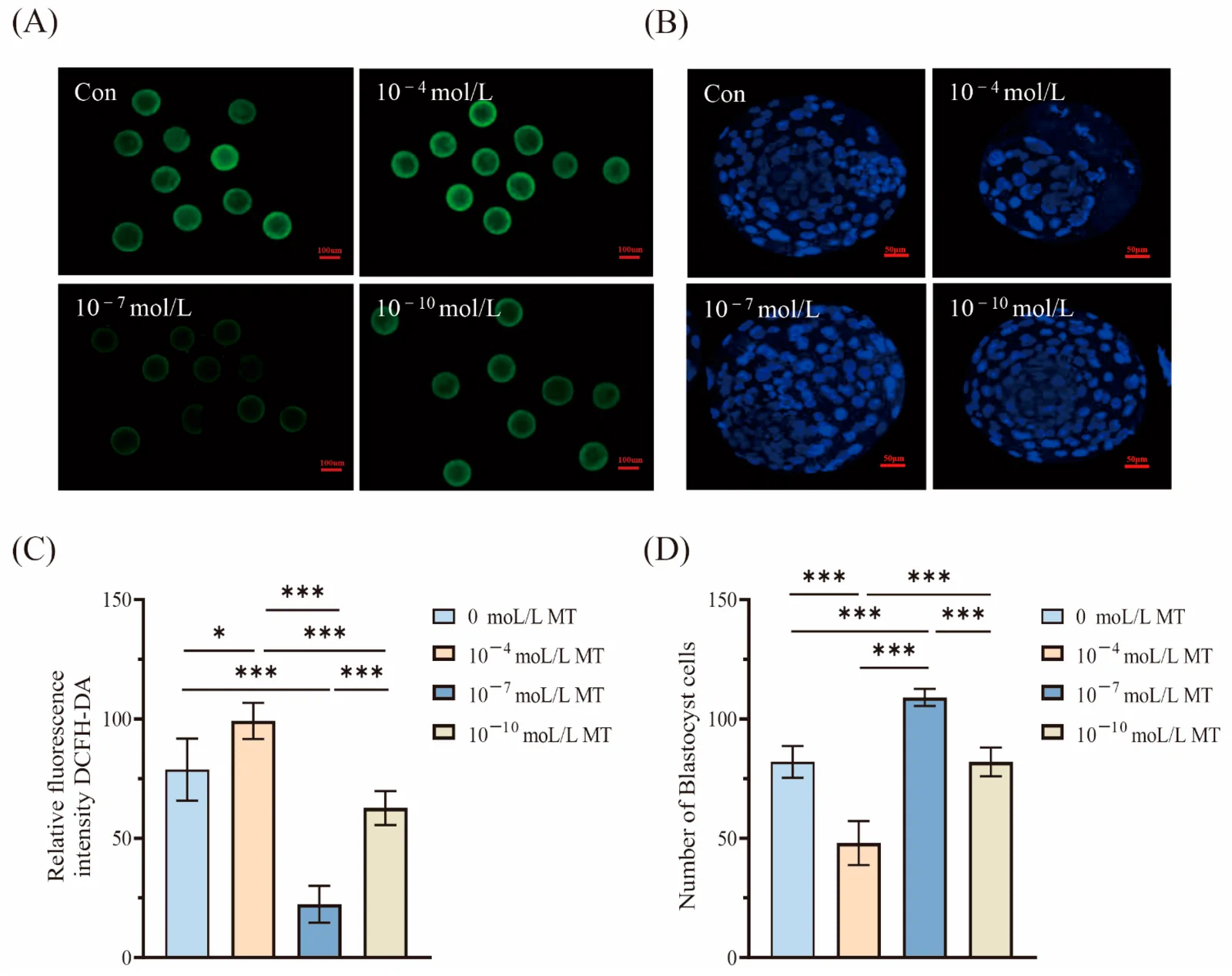
Effects of melatonin supplementation on oxidative stress in MII oocytes and subsequent blastocyst quality in bovines. (**A**). Representative fluorescence images of intracellular reactive oxygen species (ROS) levels detected by DCFH-DA staining (green). Scale bar = 100 μm. (**B**). Representative images of blastocysts with nuclear staining (blue) for total cell counting. Scale bar = 50 μm. (**C**,**D**). Statistical analysis of relative DCFH-DA fluorescence intensity (**C**) and total number of cells per blastocyst (**D**). To avoid pseudoreplication, data from individual oocytes (total 9–10 per group) or blastocysts (3 per group per run; total 12 per group) were averaged within each independent in vitro production (IVP) run to generate run-level means. Statistical analyses were performed based on these independent run-level means (*n* = 4 independent runs). * *p* < 0.05, *** *p* < 0.01.

The results from the DCFH-DA staining in MII stage oocytes showed that supplementation with 10^−7^ mol/L MT significantly decreased the ROS fluorescence intensity compared with the control and the other MT-treated groups (*p* < 0.05) (Figure 6C). Conversely, the 10^−4^ mol/L MT treatment significantly increased ROS accumulation compared to all other groups. Furthermore, DAPI staining of the resulting blastocysts (Figure 6B) demonstrated that the 10^−7^ mol/L MT group had a significantly higher total cell number per blastocyst than the control and other MT groups (*p* < 0.001) (Figure 6D). In contrast, the 10^−4^ mol/L MT group exhibited the lowest cell count among all groups, confirming its deleterious effect on embryo viability.

### 3.7. The Potential Molecular Mechanisms of MT in the Development of Bovine Blastocysts Evaluated by Smart-Seq2 Profiling

**Figure 7:**
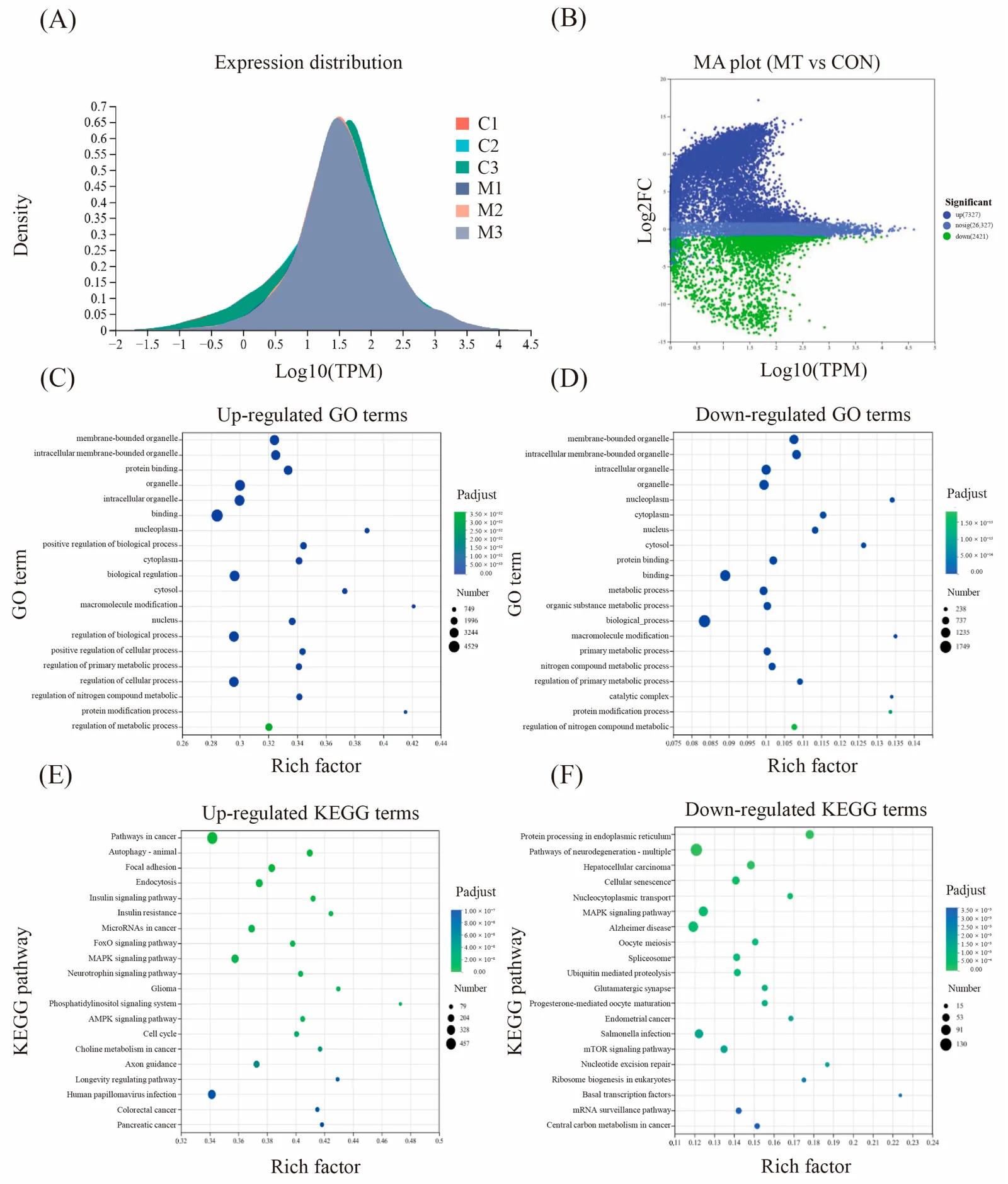
Effects of melatonin in culture medium on transcriptomic profiling in bovine blastocysts. (**A**). Density plot showing the overall gene expression distribution (log10 TPM) across the control (C1–C3) and MT-treated (M1–M3) blastocysts. (**B**). MA plot of differentially expressed genes (DEGs) between the MT and CON groups. Blue and green dots represent significantly up-regulated and down-regulated genes, respectively. (**C**,**D**). Gene Ontology (GO) enrichment analysis of the significantly up-regulated (**C**) and down-regulated (**D**) DEGs. (**E**,**F**). Kyoto Encyclopedia of Genes and Genomes (KEGG) pathway enrichment analysis of the significantly up-regulated (**E**) and down-regulated (**F**) DEGs. TPM, transcripts per million.

The results of Smart-seq2 showed that the overall gene expression distribution (log_10_ TPM) was highly consistent across all biological replicates, indicating reliable sequencing reproducibility (Figure 7A). MA plot analysis identified substantial transcriptomic alterations, with numerous differentially expressed genes (DEGs) upregulated and downregulated in the 10^−7^ mol/L MT-treated blastocysts compared to control (Figure 7B). The Gene Ontology (GO) enrichment analysis associated these DEGs predominantly with the essential cellular components and biological processes, including membrane-bound organelles, protein binding, and metabolic regulation (Figure 7C,D). KEGG pathway enrichment analysis provided deeper functional insight, i.e., the upregulated DEGs in the 10^−7^ mol/L MT group were significantly enriched in pathways vital for cellular homeostasis, energy sensing, and proliferation—notably autophagy, the FoxO signaling pathway, the AMPK signaling pathway, and the cell cycle (Figure 7E)—whereas, these downregulated DEGs were highly enriched in pathways associated with cellular stress and aging, such as protein processing in the endoplasmic reticulum (indicative of ER stress) and cellular senescence (Figure 7F). These transcriptomic signatures are consistent with the observed morphological and redox improvements of MT supplementation mentioned above.

### 3.8. Effects of MT on the Transcription of Key Genes Involved in Redox Homeostasis and Steroidogenesis In Vitro

**Figure 8:**
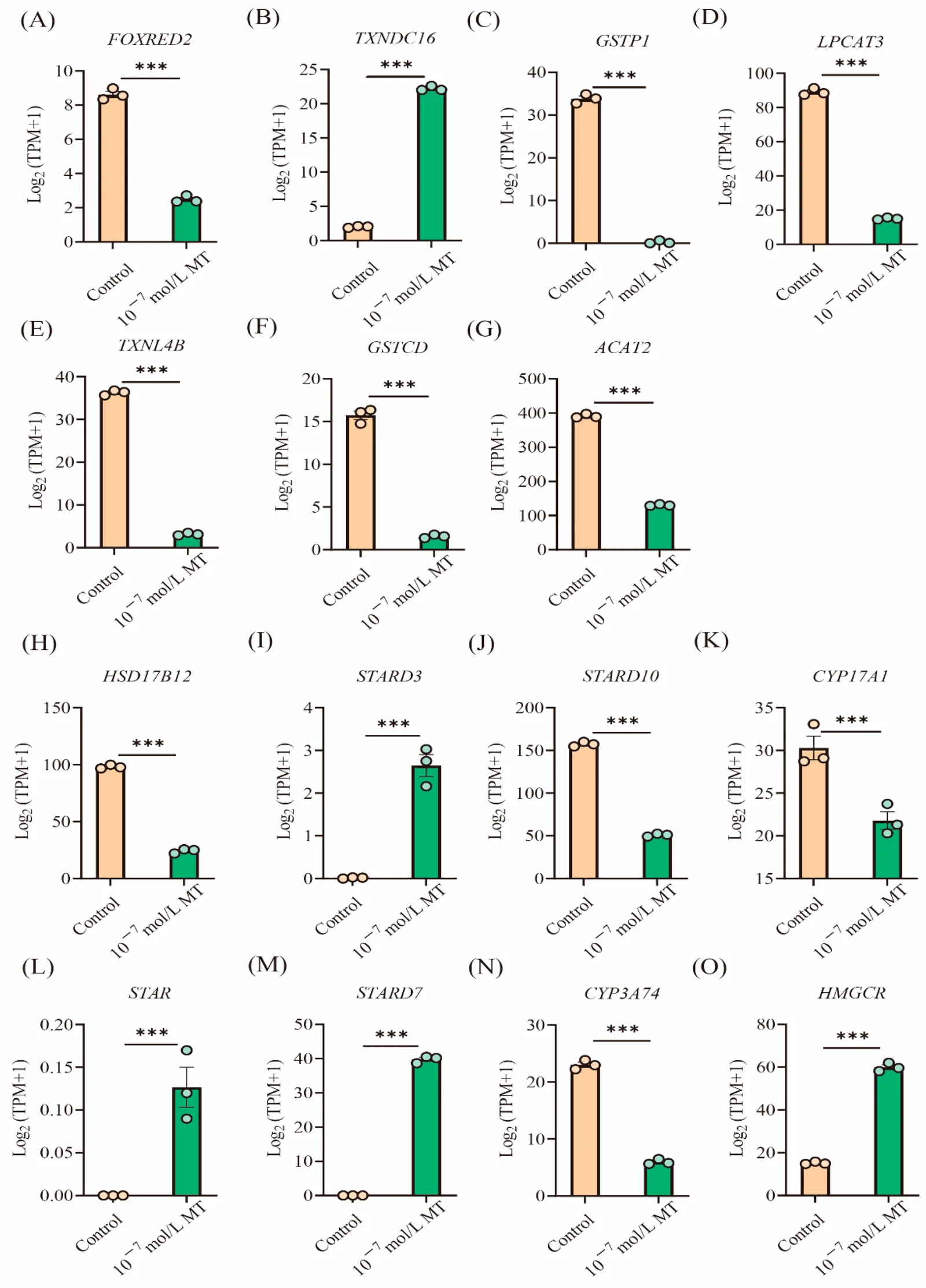
Effects of melatonin in culture medium on the expression profiles of key genes related to redox homeostasis and steroidogenesis in bovine blastocysts. (**A**–**O**). The mRNA expression levels (Log (TPM + 1)) of selected key differentially expressed genes, including *FOXRED2* (**A**), *TXNDC16* (**B**), *GSTP1* (**C**), *LPCAT3* (**D**), *TXNL4B* (**E**), *GSTCD* (**F**), *ACAT2* (**G**), *HSD17B12* (**H**), *STARD3* (**I**), *STARD10* (**J**), *CYP17A1* (**K**), *STAR* (**L**), *STARD7* (**M**), *CYP3A74* (**N**), and *HMGCR* (**O**). *TPM*, transcripts per million. *** *p* < 0.001.

To define the molecular basis of the Smart-seq2 transcriptomic shifts, the expression profiles of key regulatory genes were analyzed. Consistent with the enhanced systemic and local antioxidant capacity, *TXNDC16* was significantly upregulated (*p* < 0.001), whereas *FOXRED2*, *GSTP1*, *TXNL4B*, and *GSTCD* were significantly downregulated in the 10^−7^ mol/L MT group compared to the control (*p* < 0.001) (Figure 8A–C,E,F).

For the lipid and steroid metabolism pathways, the expression of STAR (encoding the rate-limiting transport protein for steroidogenesis), *HMGCR* (encoding the rate-limiting enzyme in cholesterol biosynthesis), and *STARD3*, as well as *STARD7* (the lipid transfer genes), were significantly upregulated (*p* < 0.001) (Figure 8I,L,M,O), whereas several other lipid and steroid metabolic genes, including *LPCAT3, ACAT2, HSD17B12, STARD10, CYP17A1*, and *CYP3A74*, were significantly downregulated (*p* < 0.001) (Figure 8D,G,H,J,K,N) in the 10^−7^ mol/L MT group compared to the control. These in vitro transcriptomic observations in blastocysts are biologically complementary to the metabolic and endocrine responses observed in the in vivo studies.

## 4. Discussion

OPU is a critical biotechnology for accelerating breeding and selective gene multiplication in dairy cattle [36,37]. The yield and developmental competence of collected oocytes from OPU serve as the primary determinants of IVEP efficiency [38,39]. The quality and quantity of oocytes are influenced by a variety of factors, including antral follicle count, donor age, breed, reproductive status, and plane of nutrition [40,41,42,43], in addition, the intensive aspiration protocols of OPU negatively impact the quality of the oocytes by inducing local and systemic oxidative stress in the donors. This oxidative stress alters the follicular microenvironment and suppresses oocyte developmental potential [44,45]. Currently, few effective methods have been identified to target the adverse effects of OPU to improve the quality, particularly regarding the local and systemic oxidative stress associated with OPU [46]. MT, as a naturally occurring molecule, exhibits a potent antioxidant capacity which is mediated in both receptor-dependent and/or receptor-independent manners [47]. The beneficial effects of MT on female reproductive physiology in cows and other mammals have been reported [48]. In vitro, its supplementation can impact the quality of the oocytes and their developmental competence [49]. However, its effects on OPU have not been systemically investigated. Thus, in the present study, by integrating the in vivo and in vitro approaches, its multiple potential protective mechanisms in an OPU model were systematically investigated.

It is well known that successful in vivo superovulation heavily relies on a stable endocrine milieu, yet OPU frequently triggers a robust stress response, characterized by elevated cortisol, that can impair gonadotropin secretion and follicular growth [50,51]. These alterations disrupt the endocrine milieu and the microenvironment for oocyte growth. Consistent with this framework, in the in vivo study, 80 mg MT per injection (cumulative dose: 320 mg) was subcutaneously injected into the cows 4 days prior to OPU at the time of each FSH injection (Figure 1B). This MT treatment significantly reduced serum cortisol and malondialdehyde (MDA) concentrations while boosting the activities of the antioxidant enzymes SOD and GSH-Px, indicating that MT blunts the oxidative stress of the follicular aspiration associated with OPU. This antioxidant activity plays an important role in improving the quality of the oocytes. In addition to the redox balance in the microenvironment of oocyte growth, the endocrine-related gonadotropic axis is also a key player. For example, FSH level is one of the most well-characterized determinants of follicle growth and dominant follicle selection. Administration of exogenous FSH stimulates continuous follicle growth and prevents follicles from becoming subordinate, allowing multiple follicles to acquire a dominant phenotype [52,53], while the pre-wave rise and subsequent nadir of endogenous FSH govern follicle recruitment and the selection of a single dominant follicle [54,55]. Treatment with 80 mg MT per injection (cumulative dose: 320 mg) in cows significantly increased their circulating levels of MT, its precursor N-acetylserotonin, and its metabolite 6-hydroxymelatonin (Figure S1). The melatonin and its metabolites were associated with lower P4 concentrations during late stimulation and sustained FSH levels. It is well known that premature elevation of progesterone is a major bottleneck in bovine superovulation, leading to an unfavorable endocrine environment, oocyte aging, and reduced fertilization capacity. The actions of melatonin, which were associated with sustained serum FSH levels and lower P4 concentrations at the measured time points, likely extend the critical window for oocyte cytoplasmic and epigenetic maturation, contributing to the increased yield of Grade A oocytes in MT-treated cows. In parallel, the elevation of endogenous MT synthesis was accompanied by a marked reduction in pro-inflammatory cytokines (IL-1α, TNF-α) and a corresponding increase in anti-inflammatory cytokines (IL-10, IL-13) (Figure S2) in this study was consistent with previous reports that MT suppressed pro-inflammatory factor release by inhibiting pathways such as NF-κB [56] while promoting anti-inflammatory cytokine secretion [57].

Beyond these beneficial effects mentioned above, MT profoundly reshaped the follicular metabolic niche. The results of the follicular metabolome showed a significant enrichment of the glutathione metabolism pathway mediated by MT. The elevated abundances of oxidized glutathione, S-lactoylglutathione, and other derivatives indicated an accelerated glutathione redox cycle. Glutathione is the most abundant endogenous antioxidant in mammalian cells and the primary defense against the ROS generated during rapid follicular growth.

MT also actively participates in follicular steroidogenesis. *STAR* is the rate-limiting transport protein responsible for translocating cholesterol into mitochondria for steroidogenesis, whereas *HMGCR* is the master rate-limiting enzyme in de novo cholesterol biosynthesis. MT upregulates STAR protein expression (around 2.5-fold) through the *cAMP/PKA/CREB* signaling pathway to promote cholesterol transport from the outer to the inner mitochondrial membrane, and increases the supply of substrates for steroid hormone synthesis by 60% [58]. At the same time, MT also significantly upregulates key cholesterol synthesis genes such as *HMGCR* (increasing mRNA levels by 2–4-fold) and increases intracellular free cholesterol content by 55% [59], in part by inhibiting *HMGCR* phosphorylation and degradation and thereby enhancing its stability. Consistent with these reports, our untargeted metabolomic analysis revealed a massive accumulation of estrogenic derivatives (such as estriol and estrone) in the follicles of MT-treated cows. Estrogen is vital for cumulus cell expansion, gap junction maintenance, and the prevention of oocyte apoptosis. This in vivo follicular metabolomic phenotype indicated that MT mobilized cholesterol substrates to generate a robust estrogenic niche highly conducive to oocyte maturation. In a biologically complementary manner, the in vitro transcriptomic data revealed a dramatic upregulation of STAR and *HMGCR* in blastocysts, reflecting a parallel modulation of steroidogenic pathways across the two distinct experimental models.

To identify the direct effects of MT on the oocytes, a cell culture was performed. This in vitro study excludes the effects of MT on the maternal system, which influences oocyte development. The in vitro findings were consistent with a previous report that adding 10^−9^ mol/L MT to the IVM medium reduced intracellular ROS, increased the blastocyst development rate from 28% to 45%, and increased the total number of blastocyst cells in the cultured bovine embryos [27]. An increased GSH/GSSG ratio, stabilized mitochondrial membrane potential, and downregulation of the pro-apoptotic gene Bax were reported when MT was added to bovine IVP systems [60]. Previous studies showed that MT scavenged ROS with twice the efficiency of vitamin E and effectively protected oocytes from lipid peroxidation and maintained DNA integrity [61]. The altered expression of key redox-regulatory genes, including TXNDC16, FOXRED2, and GSTP1, in MT-treated blastocysts further supports its antioxidant intervention on oocyte development and neutralizes the excessive ROS induced by exogenous gonadotropins and physical OPU puncture, thereby shielding the oocyte from lipid peroxidation and DNA damage. MT supplementation at the level of 10^−7^ mol/L significantly enhanced cleavage and blastocyst rates, reduced intracellular ROS accumulation, and increased the total cell number per blastocyst in cultured embryos. Notably, we observed a dose-dependent biphasic response of the cultured oocytes to melatonin; i.e., an excessively high concentration (10^−4^ mol/L) of MT exerted detrimental, pro-oxidant effects, while 10^−7^ mol/L was the most effective concentration among those evaluated for promoting bovine in vitro embryo development. Smart-seq2 transcriptomic profiling revealed that these beneficial effects of MT were associated with profound molecular reprogramming at the transcriptional level. The KEGG enrichment analysis demonstrated an enrichment of upregulated differentially expressed genes associated with the AMPK and FoxO signaling pathways, as well as autophagy-related processes, in MT-treated blastocysts compared to the control. In the literature, MT is known to activate AMPK, a cellular energy sensor, by maintaining mitochondrial membrane potential and ATP synthesis, and the activated AMPK phosphorylates the FoxO transcription factor and promotes its nuclear translocation, initiating downstream antioxidant (e.g., SOD, CAT) and autophagy gene expression and forming an “energy–stress” coupled regulatory network [62]. Previous studies have demonstrated that melatonin upregulates the expression of *ATPase 6*, *BMP-15*, *GDF-9*, *SOD-1*, *Gpx-4*, and *Bcl-2*—which are critical genes for oocyte maturation and embryo development—while downregulating the apoptotic gene *caspase-3* [63]. Our transcriptomic signatures are highly consistent with the potential modulation of this network. Conversely, transcriptomic signatures associated with endoplasmic reticulum stress and cellular senescence were significantly downregulated in MT-treated cells compared to the control. ER stress is a primary trigger of early embryonic apoptosis and developmental arrest. Thus, the observed transcriptional profiles suggest that MT appears to preserve blastocyst pluripotency and viability by potentially mitigating ER stress and suppressing senescence signatures at the transcriptional level. Therefore, the in vitro study confirmed the protective effects of melatonin on disrupted oocyte quality and embryo development in cows caused by OPU and also provided the potential mechanisms at the molecular level.

Several limitations of this study should be acknowledged. First, the work was conducted in a single breed on a single farm using nulliparous Holstein heifers within a narrow age range; therefore, the findings may not fully apply to multiparous cows, other breeds, or different management systems. Second, the follicular fluid metabolome was profiled at a single dose and a single time point, and the serum endocrine and immune measurements were limited to the per-OPU window. Future longitudinal sampling would help resolve the temporal dynamics of MT action. Third, the relationship between our in vivo and in vitro findings must be interpreted cautiously. The in vivo experiment evaluated the systemic administration of melatonin in FSH-stimulated cows, where the observed improvements in oocyte quality were likely mediated by complex systemic responses, including the modulation of hormonal dynamics and the follicular microenvironment. In contrast, the in vitro experiment evaluated the direct exposure of oocytes to melatonin, highlighting its direct cellular and antioxidant effects. While these two experimental models are highly complementary and collectively strengthen the biological argument for melatonin’s benefits, they do not constitute direct mechanistic confirmation of the exact same pathway. Finally, the metabolomic and transcriptomic analyses provide correlative insights rather than direct functional evidence. The pathways implicated here, including glutathione metabolism, STAR/HMGCR-driven steroidogenesis, and AMPK/FoxO signaling, warrant direct functional validation, for example, through receptor antagonist or gene perturbation experiments. These limitations will be addressed in future studies.

## 5. Conclusions

In the current study, by combining in vitro and in vivo experiments, we systematically investigated the protective effects of MT on disrupted oocyte quality and development caused by OPU. In the in vivo study, subcutaneous administration of 80 mg MT per injection (cumulative dose: 320 mg) to dairy cows enhanced the morphological quality and yield of recovered oocytes alongside lower circulating progesterone concentrations at the measured time points and a remodeled follicular metabolic microenvironment. These outcomes were associated with omics-derived signatures suggestive of enhanced glutathione-mediated antioxidant defenses and STAR/HMGCR-driven steroidogenesis, highlighting them as potential mechanisms. Additionally, in the in vitro experiments, direct supplementation with 10^−7^ mol/L MT improved early embryonic development and reduced intracellular ROS. Furthermore, the induced transcriptomic changes suggested a potential modulation of AMPK/FoxO-mediated stress resistance and a possible alleviation of ER stress. Importantly, while these in vivo and in vitro findings are highly complementary, they represent distinct biological contexts—systemic responses versus direct cellular effects—and do not constitute direct mechanistic confirmation of the exact same pathway. Overall, the results from these complementary studies offer a practical approach for optimizing OPU outcomes and reproductive efficiency in cattle breeding programs.

## Data Availability

The raw smart-seq 2 data were deposited in the National Center for Biotechnology Information (NCBI) (Accession number: GSE342370), which is publicly accessible at https://ncbi.nlm.nih.gov/geo/query/acc.cgi?acc=GSE342370 (accessed on 20 August 2026). Further inquiries can be directed to the corresponding authors.

## Supplementary Materials

The following supporting information can be downloaded at: https://www.mdpi.com/article/10.3390/antiox15091101/s1, Figure S1: Effects of melatonin subcutaneous injection on the serum profiles of melatonin and its metabolites in dairy cows; Figure S2: Effects of melatonin subcutaneous injection on systemic inflammation of serum cytokine profiles in dairy cows; Table S1: The differentially abundant metabolites identified using FDR-adjusted *p* < 0.05 alongside VIP and fold-change thresholds; Table S2: The principal RNA-seq quality-control metrics for each biological replicate, including sequencing depth, proportion of retained clean reads, and mapping rate; Table S3: The differentially expressed genes.
